# Challenges in the Complex Management of Neglected Cutaneous Melanomas in the Head and Neck Area: A Single Center Experience

**DOI:** 10.3390/jcm12051910

**Published:** 2023-02-28

**Authors:** Péter Lázár, Edit Tóth Molnár, Balázs Bende, Gábor Vass, Eszter Baltás, Róbert Paczona, Erika Varga, József Piffkó, Lajos Kemény, Judit Oláh, Erika Gabriella Kis

**Affiliations:** 1 Department of Maxillofacial Surgery, University of Szeged, 6720 Szeged, Hungary; 2 Department of Ophthalmology, University of Szeged, 6720 Szeged, Hungary; 3 Department of Dermatology and Allergology, University of Szeged, 6720 Szeged, Hungary; 4 Department of Oto-Rhino-Laryngology and Head & Neck Surgery, University of Szeged, 6720 Szeged, Hungary; 5 Department of Oncotherapy, University of Szeged, 6720 Szeged, Hungary

**Keywords:** melanoma, skin cancer, head and neck, giant melanoma, locally advanced, reconstructive surgery, neglected melanoma

## Abstract

Familiar controversies in the management of head and neck melanomas are more remarkable in locally advanced cases, and they represent a treatment challenge both surgically and oncologically. In our retrospective study, patients with surgically treated primary malignant melanoma of the head and neck region larger than 3 cm in diameter were included. Five patients met our inclusion criteria. In all cases, wide excision and immediate reconstruction were performed without sentinel lymph node biopsy. The defect on the scalp was covered by a split skin graft, with local flaps chosen for reconstruction on the face on an individual basis. After a 2–6 year follow-up, a good oncological, functional, and esthetic result was achieved. Our results show that in the case of large, locally advanced melanomas, surgical treatment still plays a crucial role that can provide long-term local control and support the effect of systemic treatment.

## 1. Introduction

Melanomas in the head and neck area are visible and easily recognizable for health care professionals and patients themselves; large (>3 cm) melanomas are therefore rarely encountered. Giant melanomas, which are classified as lesions larger than 10 cm in diameter, occur even more seldomly. To date, only 21 primary cutaneous giant melanomas have been described in the English-language literature; among them, eight were located on the head and neck [1,2]. Locally advanced primary melanomas of the head and neck represent a treatment challenge both surgically and oncologically and are commonly referred to a facial plastic and reconstructive surgeon.

No standard of care has been established in the management of head and neck melanomas, and clinical controversies probably exist due to the fact that large randomized clinical trials involve many other regions of the body. Large and advanced tumors especially are beyond the scope of guidelines; individualized treatment plans are therefore required from a multidisciplinary tumor board, which must comply with guideline principles. The surgery performed must meet all oncological, functional, and esthetic requirements, taking into account patient preferences as well. Fortunately, the rich vascular supply of the area provides a wide range of local reconstructive options that should be used where possible for the best cosmetic outcome [3,4,5,6].

Current treatment guidelines [7] recommend wide surgical excision with 1 to 2 cm margins depending on the Breslow thickness of the primary tumor, with the reconstruction of the defect after a final pathology report. Immediate reconstruction should only be limited to low-risk lesions and methods such as primary closure or skin grafts that can be reliably re-resected.

A sentinel lymph node biopsy (SLNB) is also recommended; however, the lymphatic mapping of the head and neck region even with “normal-size” malignant melanomas (MM) is challenging. SPECT-CT scanning is therefore advisable for the right accuracy in the head and neck region.

The aim of our study was to examine the management of large, locally advanced primary head and neck cutaneous melanomas treated at our institute in the context of current clinical standards. We were also interested in the functional and esthetic outcomes and the reasons for neglecting such apparent tumors.

## 2. Materials and Methods

In our retrospective study, a search was carried out in the database of the Department of Dermatology and Allergology, University of Szeged, between 1 January 2008, and 31 December 2019. Inclusion criteria for the study were any surgically treated primary malignant melanoma of the head and neck region larger than 3 cm in diameter. Historical data, patient characteristics, and histopathological and treatment reports were retrieved from electronic case records. The surgical treatments performed were assessed according to current NCCN practice guideline recommendations on:

1. The resection margin according to the AJCC stages [7];
2. The timing of reconstruction;
3. The sentinel lymph node biopsy.

We conducted brief interviews with the patients, their families, and two other staff members to evaluate esthetic and functional results, since there are no objective parameters to allow us to assess this type of reconstruction using uniform criteria. We therefore prepared a subjective assessment scale for both functional and esthetic outcomes, bad-acceptable-good-excellent, where excellent signifies the best result.

## 3. Results

A total of 2793 malignant melanomas were diagnosed in the period under investigation and operated on at our department; among them, 375 were in the head and neck area. Five patients (four females and one male; age: 63–92; median: 76 years) met our inclusion criteria (Table 1). In Patient 1, the primary tumor was located on the scalp and forehead; the other four patients had the melanoma on their cheek and in two cases involving the lower eyelid. The largest diameter of the lesions varied between 3 and 29 cm. The largest one (29 × 15 cm), located on the scalp, was classified as a giant melanoma. In three cases, patients only sought medical attention after distressing symptoms emerged (bleeding, disturbed vision, or an odorous tumor) despite the fact that a clinical diagnosis of melanoma had already been established in two cases 5–10 years before. Chart review revealed that medical care had been delayed because of a low level of knowledge about the nature of melanoma. Three cases involved neglect and fear of general anesthesia and medical care; however, in one case, the reason for the delay was a diagnostic failure—Patient 1 had previously received a clinical diagnosis of seborrheic keratosis. 

After melanoma was confirmed by histology from incisional biopsy samples and radiographic imaging confirmed negative nodal and general staging, wide surgical excisions of the primary tumors were performed with 1–2 cm safety margins (Table 1) with immediate reconstruction of the face, without performing SLNB in each case, based on a decision by our multidisciplinary tumor board made according to the NCCN guidelines. Tumor borders were identified, and safety margins were marked in Patients 2, 3, and 5 with dermoscopic imaging.

Average tumor thickness was 9.6 mm. After primary tumor excision, the largest diameter of the defect ranged from 4.5 to 31 cm, and the average defect size was 161.4 cm^2^, involving two to three esthetic subunits of the face. The depth of the resection on the scalp reached the periosteal layer, while that on the face in the cheek subunits was until the SMAS. In Patient 4, the entire lower eyelid from the medial to the lateral canthus was resected as the tumor was present on the conjunctiva. In Patient 5, the lower eyelid was involved superficially, so the deep part of the orbicularis oculi muscle was left intact. 

**Figure 1:**
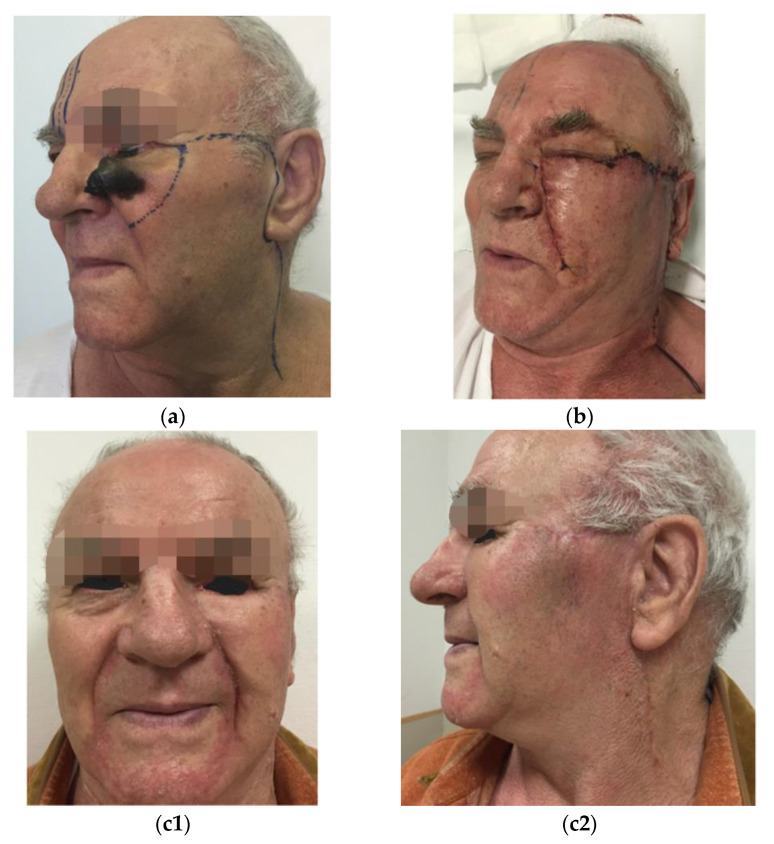
(**a**) Locally advanced NMM of the cheek preoperatively; (**b**) two-week follow up; (**c**) six-month follow-up.

**Figure 2:**
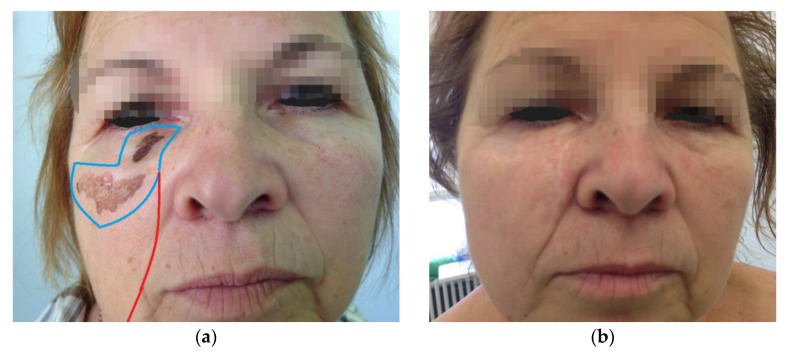

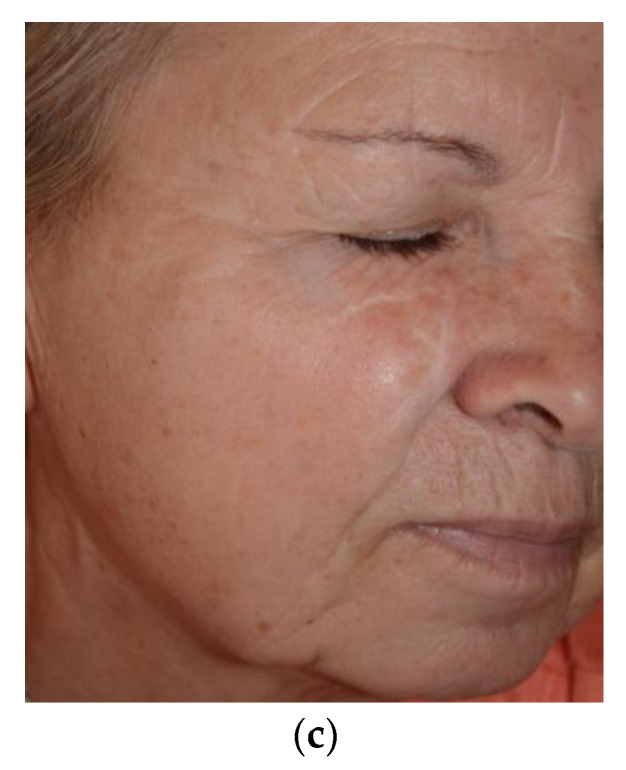
(**a**) Locally advanced LMM of the cheek preoperatively; (**b**) six-month follow up; (**c**) five-year follow-up.

In all the cases, immediate reconstructive procedures were chosen on an individual basis regarding the localization, the size of the defects, and patient age and preferences as well. The reconstruction of surgical defects were based on aesthetic units and subunits of the face for a better esthetic outcome. In two cases, in addition to the tumor removal from the cheek, the lower eyelid had to be removed either completely or partially. Therefore, in Patient 4, a modification of the Mustardé flap was used with amniotic membrane grafting in the upper part to substitute the conjunctiva (see Figure 1), and a rotation flap was performed in combination with full thickness skin grafting to the orbicularis oculi muscle in Patient 5 (see Figure 2). 

**Figure 3:**
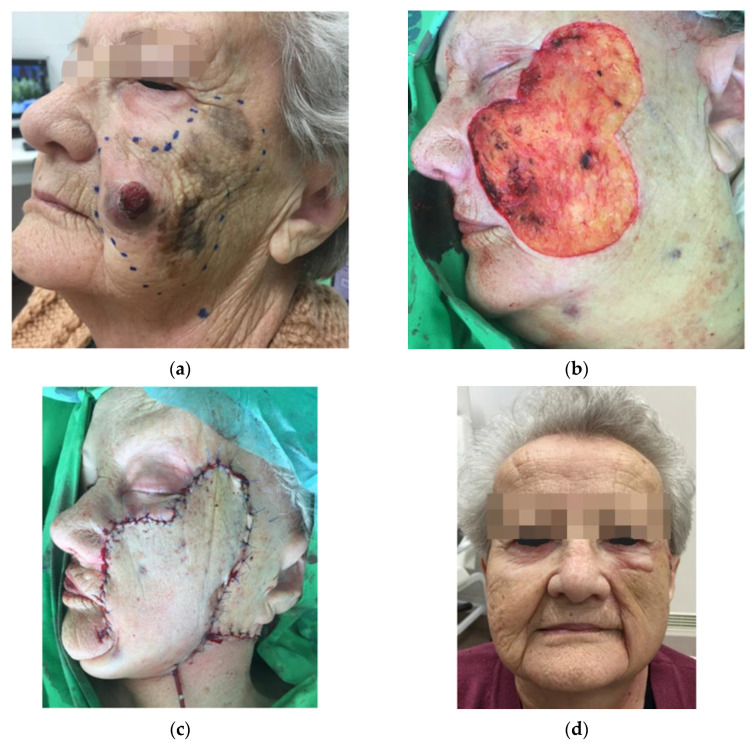
(**a**) Locally advanced LMM and NMM of the cheek preoperatively. (**b**): Defect after primary tumor excision; (**c**) immediate postoperative picture; (**d**): six-month follow-up.

In Patients 2 and 3, large cervicofacial flaps were transposed to the defect with extensive undermining on the neck and secured to the zygomatic bone by periosteal anchoring (see Figure 3).

**Figure 4:**
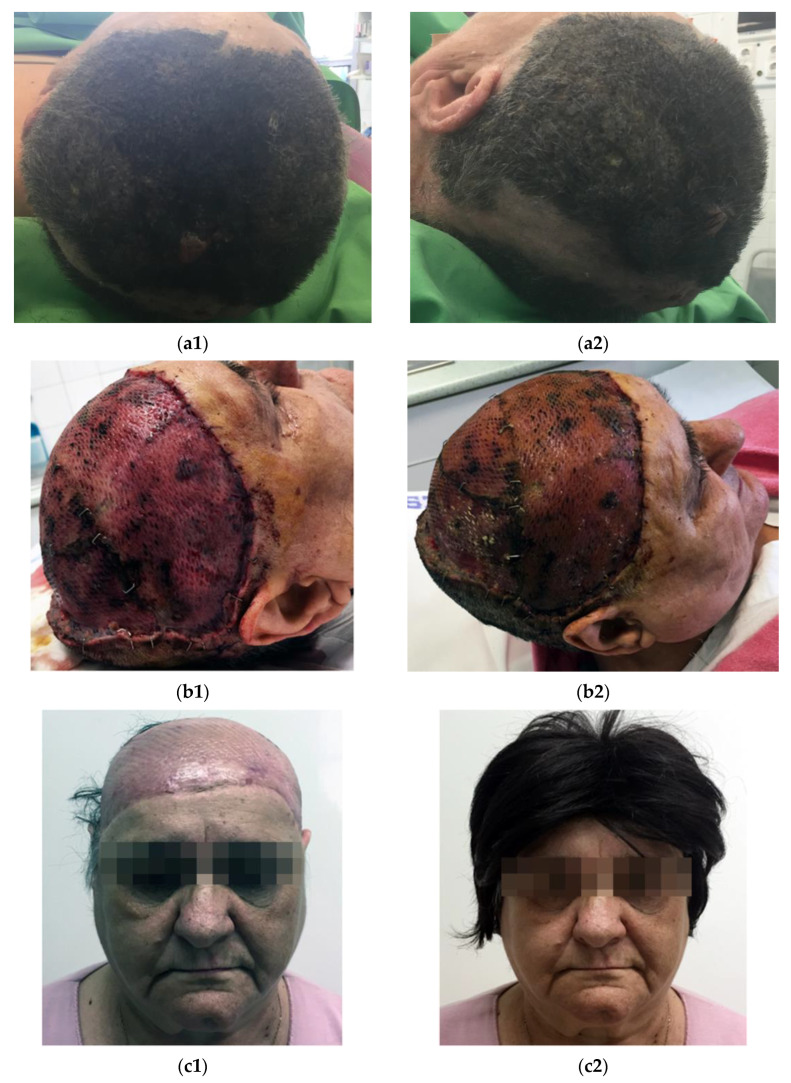
(**a**) Giant melanoma of the scalp preoperatively; (**b**) two-week follow-up, healed mesh skin graft; (**c**): six-month follow-up.

In Patient 1, after the removal of the giant melanoma from the scalp, the 627 cm^2^ defect was covered with a split-thickness skin graft harvested from the left thigh. The skin graft healed well with complete graft take (see Figure 4). 

In the early postoperative period, partial wound dehiscence was observed in two cases and a mild edema in one flap. After a median follow-up of 52 months, a good esthetic and functional result was achieved. Mimetic function was excellent in all the patients. In 4/5 patients, no local recurrence, lymphatic spread, or distant metastasis was observed in the follow-up period (29–80 months). Eight months after the primary tumor resection, Patient 1 developed multiple neck metastasis, so radical neck dissection was performed. In addition, she was enrolled in an immunotherapy clinical trial and currently has stable disease.

In terms of facial esthetics, the results were satisfactory in all the patients (Table 1). The local flaps used made it possible to maintain an adequate degree of facial harmony with similar skin coloration and texture. The final appearance of the scars was good in all the patients.

## 4. Discussion

Our report represents one of the largest series of large, locally advanced cutaneous head and neck melanomas, which is a highly variable condition and requires a specific multidisciplinary approach. Despite a great deal of ongoing research and novel therapeutic options in melanoma treatment, patients with advanced primary disease have a poor prognosis, with surgery remaining the standard of care. The goal of surgery is to remove the tumor completely, to preserve or restore normal function, and to provide the best possible cosmetic outcome.

### 4.1. Excision Margin

Current NCCN guidelines [7] recommend 1 cm margins for melanomas with a thickness of less than 2 mm and 2 cm margins for those greater than 2 mm. However, the appropriate extent of resection margins in the head and neck area is subject to debate. The use of the recommended standard margins can be difficult to obtain in this region, as it could result in severe functional and cosmetic defects or is sometimes not technically feasible at all.

Recent data has shown that resection margins of less than 1 cm increase the risk of local recurrence; however, these results are largely based on data on melanomas of other primary sites. In addition, head and neck melanomas demonstrate higher rates of recurrence and worse survival rates compared with other anatomical locations independently of margin clearance [8,9,10,11,12].

The marking of excision margins can be challenging, especially in the case of LMM; therefore, it is advised to do so with dermoscopy or confocal microscopy. Current advances in immunohistochemical staining have led to an enhanced ability to interpret frozen sections of melanoma specimens, leading to a rise in Mohs micrographic surgery in recent years. A statistically significant survival advantage was found in patients with invasive melanomas of a Breslow depth of 0.01–0.74 when treated with MMS compared with wide local excision. Despite promising results, MMS is not currently the standard of care in the treatment of invasive melanoma [13,14,15,16].

### 4.2. Management of the Neck

With a clinically node-negative neck, the surgeon has three options: the “watch and wait” policy, SLND, or elective neck dissection [17]. NCCN guideline recommends performing SLNB when the probability of positive SLND is greater than 10%–stage IB, II, or higher. Therefore, in high-risk melanomas, SLNB should always be performed when possible. In head and neck melanomas, technically it is more difficult to identify true sentinel lymph nodes (SLN-s), due to the complex and less predictable lymphatic circulation. The large size of the lesion could further limit the reliability of lymphoscintigraphic mapping. The accuracy of SLNB depends on the correct visualization and identification of true SLN-s. Therefore, in head and neck melanomas, besides lymphoscintigraphy, 3D imaging with SPECT/CT is recommended in international guidelines for better preoperative anatomical localization of SLNs [18]. If the patient is medically unfit for further treatment or unlikely to act on the information that SLNB would provide (inadequate compliance, or own wishes prevent the use of systemic treatment), then it is reasonable to forego SLNB. If SNLB cannot be performed and the patient is a candidate for possible systematic therapy, FDG-PET can be used to detect distant metastasis; however, it cannot replace SLNB due to its lower sensitivity in detecting microscopic disease. A metanalysis assessed the roles of imaging methods in melanoma (US, CT, PET-CT, and MRI); it showed that US is the best imaging method to diagnose lymph node involvement and follow-up monitoring [19,20].

Elective neck dissection at the time of primary tumor excision is not the standard of care. Two prospective RCTs have recently demonstrated no improvement in MM-specific survival or OS in patients with positive sentinel lymph node who underwent completion lymph node dissection (CLND), compared with those undergoing nodal basin ultrasonography surveillance. The significance of the SLND positivity in relation to the adjuvant systemic treatment of melanoma patients is increasing. Furthermore, administration of personally tailored innovative therapeutic agents seems to be more effective than further surgical treatment on the neck.

In the cases we have presented, close observation of the lymphatic region (every four months for two years, then every six months by ultrasonography) was decided by the multidisciplinary tumor board, with therapeutic neck dissection, if necessary. Patients with high-risk melanomas who were medically fit for further systematic treatment (Patient 1 and 4) underwent additional radiological staging and monitoring (PET-CT, CT, and MRI if required). The decision to forego SLND was made individually considering different aspects in every case: all of the patients presented with a node negative neck. In the case of Patient 1, the relevant lymphatic drainage mapping accuracy would have been questionable because the intense radioactivity of the extremely large primary tumor injection site would probably have overshadowed the visibility of the sentinel lymph nodes. Patients 2 and 3 were not medically fit for any further systematic treatment. Patient 4 refused other oncologic modalities. Finally, Patient 5 had stage IA melanoma, which required no further treatment. 

### 4.3. Immediate or Delayed Reconstruction

The decision between immediate and delayed reconstruction following melanoma excision is a risk/benefit decision that must consider disease characteristics, patient comorbidities, and planned reconstructive modality. 

Following locally advanced melanoma excision, reconstruction is historically a multistage or delayed procedure when the wound is left open until the final pathological examination has confirmed negative margins. As neither frozen section analysis nor MMS is the standard of care in treating invasive melanomas, the decision to perform immediate reconstruction is based on the likelihood of obtaining a negative margin coupled with the patient’s wishes and wound care concerns until permanent pathological results are finalized. 

In the head and neck area, positive final margins range from 5% to 12%. These cases have been associated with desmoplastic subtype, T4 stage, ulceration, and treatment of locally recurrent lesions. However, a retrospective single-institution study was only able to identify age as a predictive variable and could not associate any tumor- or treatment-related factors with positive margins [21]. For that reason, many authors recommend considering waiting for the final pathology report before performing local flap repair, even for T1 lesions.

An alternative approach is immediate reconstruction, which reduces the risks associated with multiple procedures, reduces treatment time and costs, and increases patient satisfaction, risking possible higher rates of local recurrence. Performing immediate reconstruction in the context of melanoma extirpation is debated when considering the use of local flaps. With the use of a primary closure or skin graft, in case of positive margins, the orientation of the tumor can still be discerned and re-excision can be done successfully. [22,23].

In our cases, the large defects were immediately reconstructed either with local flaps or with a skin graft. In the case of neglected tumors, in our opinion, this is the treatment of choice as patient compliance with and acceptance of the treatment are much lower in these cases than in the normal patient population. In addition, these tumors usually present in the elderly, where it is crucial to reduce treatment time, hospitalization, and risks as much as possible.

### 4.4. Function and Esthetics

In the head and neck area, reconstruction is required in approximately 13% of melanoma cases [24], which is often complicated because of the close proximity of sensitive anatomical structures. Proper flap selection and design mainly depends on the size and location of the defect and the thickness and laxity of the skin. It is not possible to set up a rigid algorithm; each case must be approached with a unique plan. When possible, local or locoregional flaps are the treatment of choice as they provide the best possible color and texture match with the surrounding skin, even if more esthetic units of the face are violated [25,26]. 

The skin on the cheek has a rich blood supply and is more mobile than other areas of the face. Here, primary closure is possible for many defects up to 2 cm in diameter. Larger defects may also be closed with advancement flaps in the elderly because of the lax skin. However, primary skin closure may be problematic in the perioral, perinasal, and periorbital regions, which may become distorted. Therefore, larger defects may be reconstructed with skin grafts, but this solution may yield poor esthetic results due to the color and texture mismatch at the grafted site (see Figure 4).

A variety of local skin flaps makes it possible to reconstruct superficial defects larger than 2 cm. The mixed rotational and advancement Mustardé flap is suitable for defects that are located in the medial cheek and zygomatic areas and can be oriented within lines of skin tension.

The arc of the flap is designed high in the temporal area to allow for ample medial advancement (see Figure 1).

Other flaps include rhomboid and Esser flaps. These flaps transpose adjacent tissue and are most useful for defects that do not exceed 4 cm. They may result in abnormal contour and visible scarring because donor site scars are difficult to place in lines of skin tension (see Figure 2).

Cervicofacial flaps are larger advancement/rotation flaps which are used to reconstruct superficial defects that exceed 6 to 10 cm in size. These flaps recruit skin from the adjacent cheek and neck and provide a good contour and color match (see Figure 3).

Extensive undermining is necessary to allow adequate mobilization and prevent tension along the incisions. The blood supply to these large flaps is from multiple cervical and clavicular perforators. Flaps that recruit skin from the neck may incorporate platysma muscle to improve blood supply to the distal flap tissues. The skin used had characteristics identical to those of the affected tissue. In the older patients, the volume provided by the flap was sufficient even for very large defects, so more complex, higher-morbidity alternative techniques were not considered.

### 4.5. Why Is a Tumor Neglected? 

It is rare for patients to present with such advanced lesions in the head and neck area as these tumors are easily recognizable for the patients themselves and such advanced lesions even hinder social interactions. Factors leading to patients delaying seeking medical care are not always clear but could include the pursuit of alternative medicine, low level of knowledge about skin tumors, socioeconomic factors, underlying diseases, including psychiatric conditions, and fear (of illness or medical care) as well as diagnostic failure. Fear of medical care is a serious problem, especially in elderly patients, resulting in serious symptoms, e.g., bleeding, disturbed vision, or an odorous tumor, when patients present themselves. It is important to respect these patients’ wishes and limitations and tailor treatment accordingly. 

Surgical therapy plays an important role in treatment as it is fast, effective, and potentially curative. With a rapid result that is also noticeable as such for patients, we can gain their trust and faith in recovery, so that they agree to further systemic treatment, which they would otherwise not have done in the first place. It should also be noted that in most cases, melanomas on the face do not show a BRAF mutation (the histology type is usually LMM), thus limiting treatment options, and often a patient’s comorbidities or lack of compliance prevent the use of systemic treatment altogether [27]. In these cases, the role of surgical treatment becomes even more important. Radiotherapy may be an alternative as a palliative or adjuvant treatment, but patients tend not to choose it because it lasts longer.

## 5. Conclusions

Our report represents one of the longest series of large, locally advanced cutaneous head and neck melanomas, a highly variable condition requiring a specific multidisciplinary approach. Despite the tremendous amount of research on systemic therapeutic options, surgical treatment still plays a crucial role and can provide good local control in surgically demanding localizations as well. In the case of large, advanced primary MMs, a wide local excision and immediate reconstruction are acceptable. SLNB should be performed when possible; however, PET-CT is recommended for staging and follow-up monitoring when it is not feasible for technical, anatomical, or patient-specific reasons. In our cases, local flaps provided a simple solution for the repair of large cutaneous facial defects with good esthetic and functional results and low morbidity. 

## Figures and Tables

**Table 1:** Data summary of included patients.

Patients	Gender	Age	Tumor size	Location/ Cosmetic Units Involved [8]	Histopathology Ulceration (U)	AJCC Stage	Reconstruction/ Defect Size	Resection Margins and Clearance	Other Interventions/Treatments	Follow-Up	Esthetic Result
1	F	68	29 × 15 cm	Scalp + Forehead 1b;9	NM Cl V. B:10.336 mm pT4b U+	IIID	Split skin graft/ 33 × 19 cm–627 cm^2^	2 cm–negative	Radical neck dissection BRAF-MEK inhibitor +PD1 inhibitor	52 mo. stable disease	Accept-able
2	F	92	9 × 7.6 cm	Cheek 4a,b;5b	LMM + NM Cl V. B: 17.860 mm PT4b U+	IIc	Transpo-sitional flap, Cervico-facial flap/11 × 9.6 cm–105 cm^2^	1 cm–negative	—	50 mo. tumor free	Good
3	F	92	2 × 3 cm	Cheek 4a,b,c	LMM Cl III. B: 2.736 mm pT3a U-	IIa	Cervico-facial flap/ 4 × 5 cm–20 cm^2^	1 cm–negative	—	29 mo. tumor free	Good
4	M	76	3 × 1.5 cm	Cheek + Lower eyelid 4a, 3b	LMM + NM Cl V. B: 17.024 mm pT4b U+	IIc	Extended Mus-tardé flap + amnion mem-brane graft/7 × 5.5 cm–38.5 cm^2^	2 cm–negative	—	52 mo. tumor free	Good
5	F	63	3 × 1.3 cm	Cheek + lower eyelid 3b,4a,b	LMM + NBCC Cl II. B: 0.152 mm PT1a U-	Ia	Rotation flap + full thickness skin graft/5 × 3.3 cm–165 cm^2^	1 cm–negative	—	80 mo. tumor free	Excellent

## Data Availability

Not applicable.
